# Weak-Light Phase-Locking Time Delay Interferometry with Optical Frequency Combs

**DOI:** 10.3390/s22197349

**Published:** 2022-09-28

**Authors:** Mingyang Xu, Hanzhong Wu, Yurong Liang, Dan Luo, Panpan Wang, Yujie Tan, Chenggang Shao

**Affiliations:** 1 MOE Key Laboratory of Fundamental Physical Quantities Measurement, Hubei Key Laboratory of Gravitation and Quantum Physics, National Precise Gravity Measurement Facility, School of Physics, Huazhong University of Science and Technology, Wuhan 430074, China; 2 State Key Laboratory of Applied Optics, Changchun Institute of Optics, Fine Mechanics and Physics, Chinese Academy of Sciences, Changchun 130033, China

**Keywords:** weak-light phase-locking, optical frequency comb, time delay interferometry

## Abstract

In the future space-borne gravitational wave (GW) detector, the optical transponder scheme, i.e., the phase-locking scheme, will be utilized so as to maintain the signal-to-noise ratio (SNR). In this case, the whole constellation will share one common laser equivalently, which enables the considerable simplification of time delay interferometry (TDI) combinations. Recently, and remarkably, the unique combination of TDI and optical frequency comb (OFC) has shown a bright prospect for the future space-borne missions. When the laser frequency noise and the clock noise are synchronized using OFC as the bridge, the data streams will be reasonably simplified. However, in the optical transponder scheme, the weak-light phase-locking (WLPL) loops could bring additional noises. In this work, we analyze the phase-locking scheme with OFC and transfer characteristics of the noises including the WLPL noise. We show that the WLPL noise can be efficiently reduced by using the specific TDI combination, and the cooperation of phase-locking and frequency combs can greatly simplify the post-processing.

## 1. Introduction

The direct observatory of the gravitational waves can provide a new and powerful approach to investigate the universe and the new physics. In 2016, the Laser Interferometer Gravitational-Wave Observatory (LIGO) successfully detected the first event of GWs in the frequency band from tens of Hz to several kHz [1,2]. Different from the ground-based GW detectors, the future space-borne GW detectors [3,4,5] will focus on the GWs in the lower band from 0.1 mHz to 1 Hz, which is able to cover more GW sources. In general, the space-borne GW detector is composed of a huge triangle constellation with $10^{8}$–$10^{9}$ m arm length. Two drag-free proof masses are housed in each spacecraft, and the GW signals can softly change the relative distance between the proof masses in two adjacent spacecraft. Optical interferometry will be exploited to precisely sense this length change by using Fabry–Perot-stabilized lasers. In practice, the scheme of the optical transponder will be utilized in order to enhance the optical power in the laser links and to maintain the SNR. In this case, one laser serves as the master, and all the other lasers are locked to this master laser under a specific frequency plan.

Since the GWs are often extremely weak at about $10^{-20}/{\mathrm{Hz}}^{1/2}$, the suppression of various noises is of great importance. In the future space-borne GW detectors, the technique of TDI [6,7,8] will be utilized, aiming to effectively reduce the laser frequency noise and the clock noise. Since the first demonstration, TDI has been developed for over twenty years, and various TDI combinations have been proposed in the post-processing, whose performance can well satisfy the requirement of the GW detection. The basis of TDI is to establish a virtual equal-arm interferometer by time shifting and recombining the data streams, so that the laser frequency noise can be aligned and removed while the GW signals can be preserved. In the reduction of the clock noise, the conventional strategy exploits the electro-optic modulation to generate two sidebands around the laser carrier [9,10,11]. Hereafter, the sidebands will take along the clock noise, and be transferred to the distant spacecraft. By virtue of the carrier–carrier beat and the sideband–sideband beat, the laser frequency noise and the clock noise can be reduced by using multi steps of TDI combinations. The alternative way to cancel out the clock noise is to use the OFCs to link the laser and the clock, and the sideband modulation is thus not required. Optical frequency combs have found a number of applications in the past two decades [12]. Recently, frequency-comb-based TDI has been reported both theoretically [13] and experimentally [14], and shows a bright prospect in space-borne missions. The comb lasers consist of a series of coherent lines in the frequency domain, and each line can be expressed as $N\times f_{\mathrm{rep}}+f_{\mathrm{ceo}}$. *N* is an integer, $f_{\mathrm{rep}}$ is the repetition frequency, and $f_{\mathrm{ceo}}$ is the carrier-envelope-offset frequency. In the frequency-comb-based TDI, one stable optical reference can be downconverted into the microwave region, which is able to work as the frequency reference [15].

As mentioned before, the inter-spacecraft optical transponder will be used in the future space-borne GW detectors. In practice, the power of the incoming beam in the spacecraft is often very weak at pW level (e.g., 100 pW) due to the beam divergence. This means that the phase-locking loop could suffer from such low optical power, and introduce the additional noises. In the past years, TDI with optical transponder has been investigated in great depth, and the TDI combinations can be simplified [16,17]. In spite of this, the phase-locking noises due to the weak light have never been considered. Generally speaking, the WLPL noises are related to the phase measurement noise, the photodetector noise, the weak signal noise, the laser phase noise, etc. [18,19]. Now, the performance in the higher frequency band can already reach the limit of the shot noise, but in the lower frequency band there is still the residual noise possibly due to the thermal effects or the electrical noises [20,21,22]. It is not easy work to reach the shot noise limit in the whole band from 0.1 mHz to 1 Hz in the case of the WLPL. We consider that it is possible and necessary to develop the TDI combinations capable of reducing the WLPL noise in the post-processing. Now, TDI with frequency combs is attracting increasing interesting in the field of the space-borne GW detection. However, frequency-comb-based TDI with the optical transponder has not been described, and, further, the reduction of the WLPL noise has not been demonstrated either.

In this work, we derive the frequency-comb-based TDI combinations with the optical transponder. The laser frequency noise and the clock noise can be coherently linked by using frequency comb, and therefore the TDI combinations can be further simplified. We consider that the WLPL noise can be reduced with the help of the data stream of the error signal in the locking loop. Finally, we perform the time-domain simulation to examine the performance of the presented TDI combination.

## 2. Architecture of the Space-Borne Optical Interferometer

**Figure 1:**
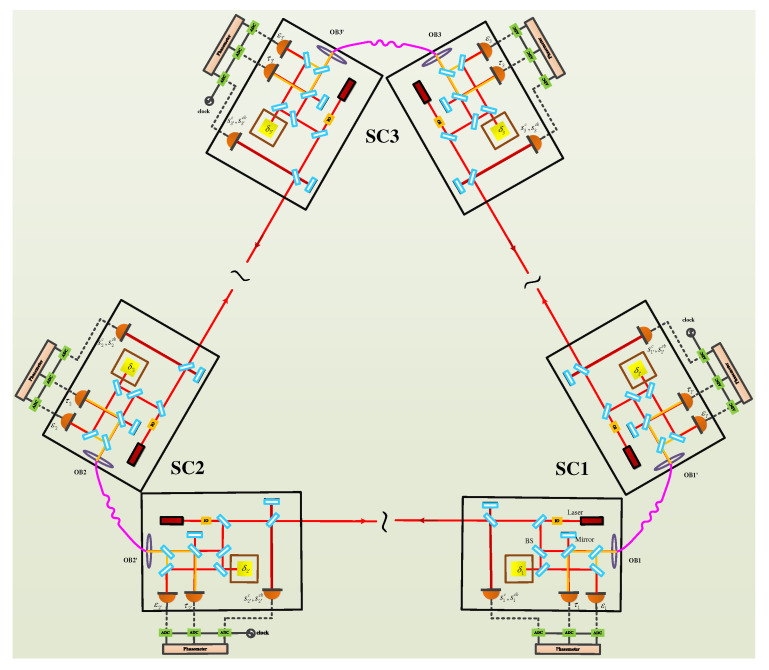
Optical and electrical setup of the inter-spacecraft.

**Figure 2:**
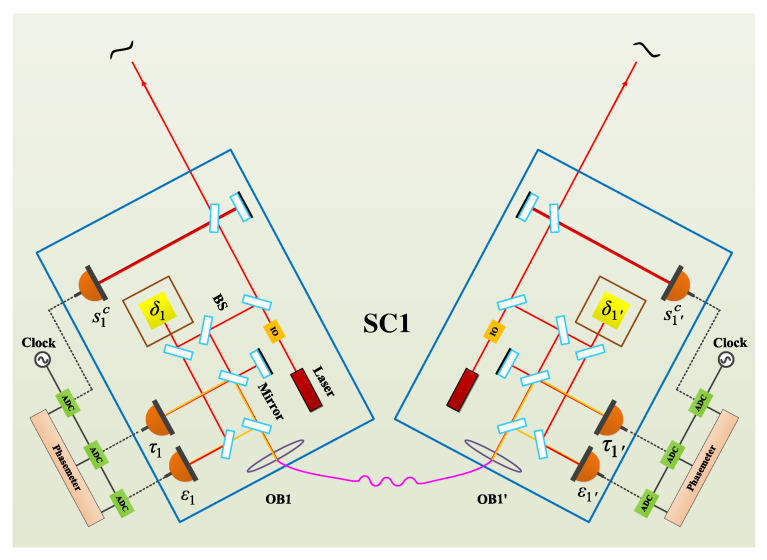
Detailed setup from one of the spacecrafts. BS: beam splitter; SC: spacecraft; OB: optical bench.

The space-borne GW detector is based on the optical interferometry, which involves a huge equilateral triangle, as shown in Figure 1. Inside the spacecraft, there are two sets of optical interferometry systems, and each system contains one laser, one proof mass, one ultra-low expansion (ULE) bench, and three phase measurements (corresponding to the scientific data stream, the reference data stream, and the proof mass data stream), as shown in Figure 2. In the post-data process, TDI will be exploited to reduce the noises using these data streams.

We suggest the expressions in Ref. [23], in which the subscript *i* corresponds to the beam reaching the spacecraft *i*, and primed or unprimed represents that the beam is propagating clockwise (CW) or counterclockwise (CCW). We also adopt $D_{i}$ and $D_{i^{\prime }}$ as the time-delay operators, which satisfy the abbreviation rule $D_{i^{\prime }}D_{i}p\left(t\right)=D_{i^{\prime }i}p\left(t\right)$. p(t) is the laser frequency noise. Please note that the speed of light in a vacuum is assumed to be unity in this work. First, we present an open-loop data stream in SC1 which means there is no phase lock, and the remaining measurements can be obtained by cyclic permutation of the spacecraft indices.

The four open-loop measurements from optical bench 1 which are similar to optical bench $1^{\prime }$:

(1) $$\begin{array}{cc} s_{1}^{c}= & h_{1}+D_{3}p_{2^{\prime }}-p_{1}+\left({\vec{n}}_{3}\cdot D_{3}{\vec{\Delta }}_{2^{\prime }}+{\vec{n}}_{3^{\prime }}\cdot {\vec{\Delta }}_{1}\right)-a_{1}q_{1}+N_{1}^{opt}, \end{array}$$

(2) $$\begin{array}{cc} \varepsilon_{1}= & p_{1^{\prime }}-p_{1}-2{\vec{n}}_{3^{\prime }}\cdot \left({\vec{\delta }}_{1}-{\vec{\Delta }}_{1}\right)+\mu_{1}-b_{1}q_{1}, \end{array}$$

(3) $$\begin{array}{cc} \tau_{1}= & p_{1^{\prime }}-p_{1}+\mu_{1}-b_{1}q_{1}, \end{array}$$

where $s_{i}$, $s_{sb}$, $\varepsilon_{i}$, and $\tau_{i}$ are the inter-spacecraft carrier-to-carrier, sideband-to-sideband measurement, the proof mass-to-optical bench, and bench-to-bench measurements, respectively. $h_{i}$ is the GW signal. $D_{i}$ is the time-delay operator. $p_{i}$, $q_{i}$, $n_{i}$, ${\vec{\Delta }}_{i}$, ${\vec{\delta }}_{i}$, $N_{i}^{opt}$, and $\mu_{i}$ are the laser frequency noise, clock noise, unit vectors between spacecraft, spacecraft motion noise, proof mass noise, shot noise, and the fiber noise. $a_{i}$, $b_{i}$, $c_{i}$, and are the coefficients corresponding to the heterodyne frequency. Assuming the unit vectors between spacecraft are positive in the counterclockwise direction, we have ${\vec{n}}_{\left(i+1\right)}\cdot {\vec{\Delta }}_{i^{\prime }}=\Delta_{i^{\prime }}$ and ${\vec{n}}_{{\left(i-1\right)}^{\prime }}\cdot {\vec{\Delta }}_{i}=-\Delta_{i}$. These data streams can be linearly combined with delays determined by the pseudorandom noise (PRN) ranging or TDI ranging [24], so that the noises can be aligned in the time domain. After several steps of combinations, the noises can be well canceled out, while the GW signals can be retained.

## 3. Phase-Locking Schemes in the Constellation

**Figure 3:**
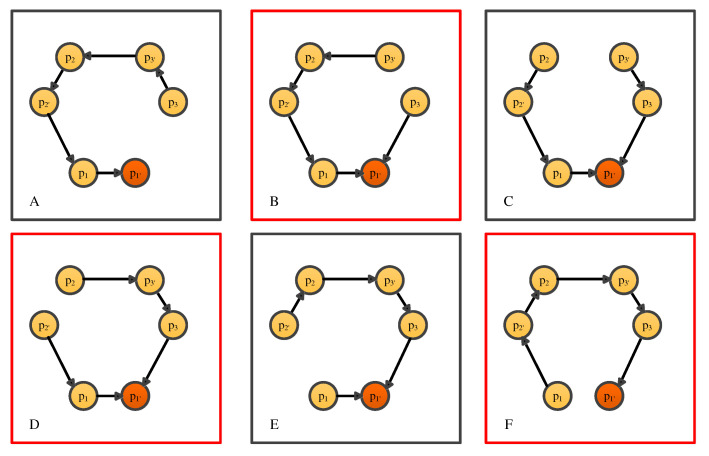
Six different phase-locking schemes.

In the optical interferometer of the Laser Interferometer Space Antenna (LISA), the optical transponder scheme, i.e., the phase-locking scheme, will be adopted. In LISA, there will be six lasers, and one laser is the master laser. If laser $1{}^{\prime }$ (i.e., the laser on optical bench $1{}^{\prime }$) is the master laser, we can derive six different phase-locking schemes, schemes A, B, C, D, E, and F, as shown in Figure 3.

(4) $$\begin{array}{c} A:\underline{p_{1^{\prime }}}\leftarrow p_{1}\leftarrow p_{2^{\prime }}\leftarrow p_{2}\leftarrow p_{3^{\prime }}\leftarrow p_{3}, \end{array}$$

(5) $$\begin{array}{c} B:p_{3}\to \underline{p_{1^{\prime }}}\leftarrow p_{1}\leftarrow p_{2^{\prime }}\leftarrow p_{2}\leftarrow p_{3^{\prime }}, \end{array}$$

(6) $$\begin{array}{c} C:p_{3^{\prime }}\to p_{3}\to \underline{p_{1^{\prime }}}\leftarrow p_{1}\leftarrow p_{2^{\prime }}\leftarrow p_{2}, \end{array}$$

(7) $$\begin{array}{c} D:p_{2}\to p_{3^{\prime }}\to p_{3}\to \underline{p_{1^{\prime }}}\leftarrow p_{1}\leftarrow p_{2^{\prime }}, \end{array}$$

(8) $$\begin{array}{c} E:p_{2^{\prime }}\to p_{2}\to p_{3^{\prime }}\to p_{3}\to \underline{p_{1^{\prime }}}\leftarrow p_{1}, \end{array}$$

(9) $$\begin{array}{c} F:p_{1}\to p_{2^{\prime }}\to p_{2}\to p_{3^{\prime }}\to p_{3}\to \underline{p_{1^{\prime }}}. \end{array}$$

**Figure 4:**
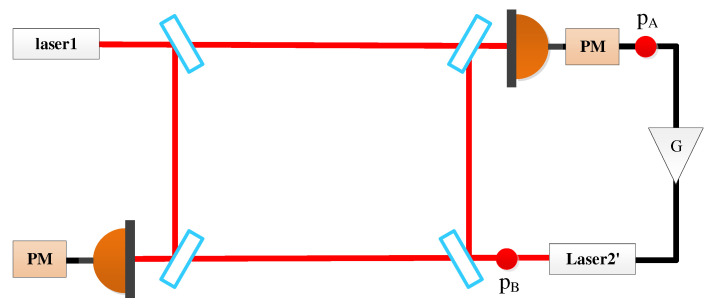
Simplified schematic of the LISA measurement scheme with phase-locking.

There are two types of phase-locking in the constellation: inner-spacecraft and inter-spacecraft locking, where the inter-spacecraft locking is with weak light. Figure 4 shows the schematic of the inter-spacecraft locking, and only two lasers are involved for simplicity.

To start with, when G = 0 (open loop), the signal at $p_{A}$ point in the frequency domain is

(10) $$\begin{array}{cc} p_{A}^{OL}\left(s\right)= & e^{-s\tau_{3\prime }}p_{1}\left(s\right)-p_{2^{\prime }}\left(s\right)\\ \; & +\left[-e^{-s\tau_{3\prime }}\Delta_{1}\left(s\right)+\Delta_{2^{\prime }}\left(s\right)\right]-a_{2^{\prime }}q_{2}\left(s\right)+N_{2^{\prime }}^{opt}{(s)+h}_{2^{\prime }}\left(s\right), \end{array}$$

where $\tau_{3^{\prime }}$ is the time delay between spacecraft 1 and 2. If the phase-locking loop (PLL) is closed, the signal at $p_{B}$ point is

(11) $$\begin{array}{cc} p_{B}^{CL}\left(s\right)= & p_{2^{\prime }}\left(s\right)+G\left(s\right)\left\{e^{-s\tau_{3\prime }}p_{1}\left(s\right)-p_{B}\left(s\right)+\left[-e^{-s\tau_{3\prime }}\Delta_{1}\left(s\right)+\Delta_{2^{\prime }}\left(s\right)\right]-a_{2^{\prime }}q_{2}\left(s\right)+h_{2^{\prime }}\left(s\right)+N_{2^{\prime }}^{opt}\left(s\right)\right\}\\ = & \left[e^{-s\tau_{3^{\prime }}}p_{1}\left(s\right)+\left(-e^{-s\tau_{3^{\prime }}}\Delta_{1}\left(s\right)+\Delta_{2^{\prime }}\left(s\right)\right)-a_{2^{\prime }}q_{2}\left(s\right)+h_{2^{\prime }}\left(s\right)+N_{2^{\prime }}^{opt}\left(s\right)\right]\\ \; & -\frac{1}{1+G(s)}\left\{e^{-s\tau_{3^{\prime }}}p_{1}\left(s\right)-p_{2^{\prime }}\left(s\right)+\left[-e^{-s\tau_{3^{\prime }}}\Delta_{1}\left(s\right)+\Delta_{2^{\prime }}\left(s\right)\right]-a_{2^{\prime }}q_{2}\left(s\right)+h_{2^{\prime }}\left(s\right)+N_{2^{\prime }}^{opt}\left(s\right)\right\}. \end{array}$$

We can write Equation (11) in the time domain with inverse Laplace transform, which is

(12) $$\begin{array}{c} p_{B}^{CL}\left(t\right)=\left\{D_{3^{\prime }}p_{1}\left(t\right)+\left[-D_{3^{\prime }}\Delta_{1}\left(t\right)+\Delta_{2^{\prime }}\left(t\right)\right]-a_{2^{\prime }}q_{2}\left(t\right)+h_{2^{\prime }}\left(t\right)+N_{2^{\prime }}^{opt}\left(t\right)\right\}+N_{2^{\prime }}^{PLL}\left(t\right). \end{array}$$

We find that when the PLL is closed, several noises, including the spacecraft motion noise, clock noise, shot noise, and the WLPL noise, are involved in the slave laser, in addition to the frequency noise of the master laser. In this work, we focus on the WLPL noise, i.e., the last term in Equation (12). Comparing Equation (12) with Equation (11), the WLPL noise can be expressed as

(13) $$\begin{array}{cc} N_{2^{\prime }}^{PLL}\left(t\right)= & L^{-1}\{-\frac{1}{1+G(s)}[e^{-s\tau_{3^{\prime }}}p_{1}\left(s\right)-p_{2^{\prime }}\left(s\right)\\ \; & +\left[-e^{-s\tau_{3^{\prime }}}\Delta_{1}\left(s\right)+\Delta_{2^{\prime }}\left(s\right)\right]-a_{2^{\prime }}q_{2}\left(s\right)+h_{2^{\prime }}\left(s\right)+N_{2^{\prime }}^{opt}\left(s\right)]\}. \end{array}$$

We find that the WLPL noise is related to the laser frequency noise of laser 1 and laser $2^{\prime }$, the SC motion noise, the clock noise, the shot noise, and the gain of the locking loop. If the gain is sufficiently large, the dominating source would be $p_{2^{\prime }}$ with the factor of 1/(1+G), since the laser $2^{\prime }$ is initially free.

Please note that the WLPL noise can be measured by the error signal in the locking loop, i.e., the signal at $p_{A}$, which can be expressed as

(14) $$\begin{array}{cc} p_{A}^{CL}\left(s\right)= & e^{-s\tau_{3\prime }}p_{1}\left(s\right)-p_{B}^{CL}\left(s\right)+\left[-e^{-s\tau_{3\prime }}\Delta_{1}\left(s\right)+\Delta_{2^{\prime }}\left(s\right)\right]-a_{2^{\prime }}q_{2}\left(s\right)+N_{2^{\prime }}^{opt}{(s)+h}_{2^{\prime }}\left(s\right)\\ = & \frac{1}{1+G(s)}\left\{e^{-s\tau_{3^{\prime }}}p_{1}\left(s\right)-p_{2^{\prime }}\left(s\right)+\left[-e^{-s\tau_{3^{\prime }}}\Delta_{1}\left(s\right)+\Delta_{2^{\prime }}\left(s\right)\right]-a_{2^{\prime }}q_{2}\left(s\right)+h_{2^{\prime }}\left(s\right)+N_{2^{\prime }}^{opt}\left(s\right)\right\}. \end{array}$$

**Figure 5:**
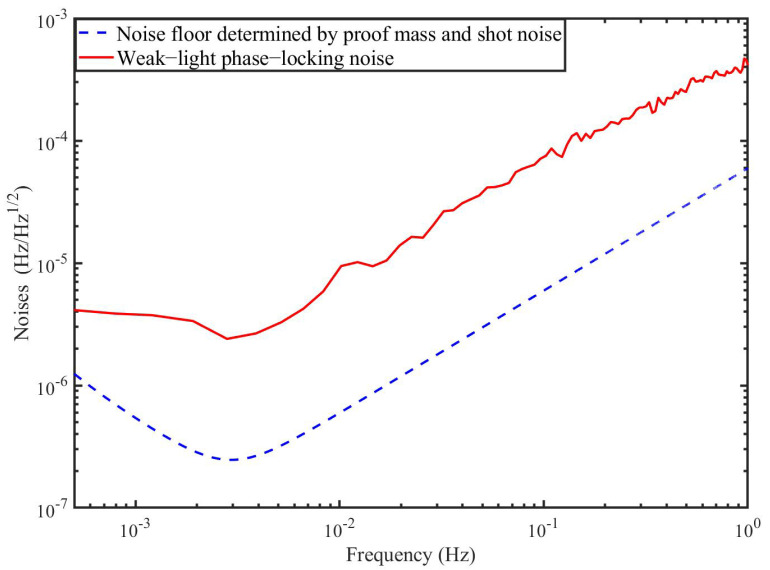
A measurement of the WLPL noise (solid trace), and the sensitivity of the GW detection (dashed trace).

In fact, we measured the WLPL noise in our lab when the optical power of the master laser (1064 nm) is 100 pW. The result is shown in Figure 5, and the WLPL noise is above the noise floor determined by the proof mass noise and the shot noise.

## 4. Phase-Locking TDI Combinations with OFCs

**Figure 6:**
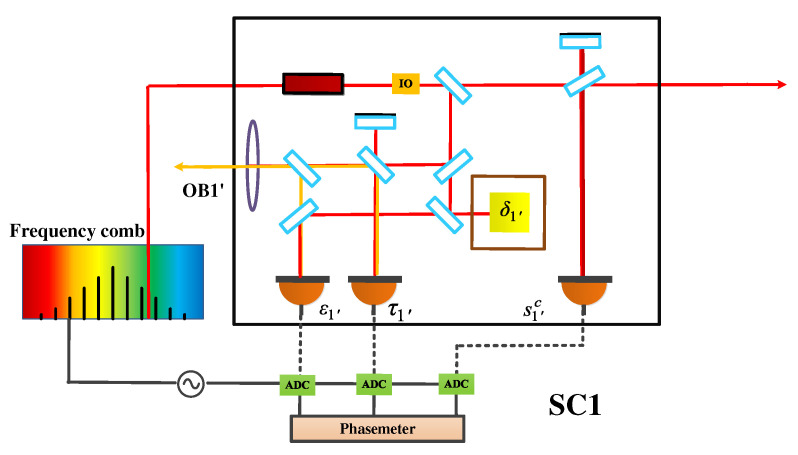
Space-borne optical interferometer with OFC.

Several nice reviews can be found for the fundamentals and the applications of OFCs [12,25,26]. In general, a few mechanisms are capable of generating frequency combs, such as mode-locked techniques [27], electro-optic modulation [28], and Kerr effect in microresonators [29]. Recently, combs based on the microresonators, also referred to as microcombs, are standing at the frontiers of the field due to their compact footprints, low power consumption, and high repetition rate [30]. We consider that microcombs can be qualified candidates for future space missions. Generally speaking, two methods can be used to realize this optic-to-microwave link. One is to stabilize one specific line of the comb to the optical reference by feedback controlling $f_{\mathrm{rep}}$, while $f_{\mathrm{ceo}}$ is locked to another reference. Consequently, $f_{\mathrm{rep}}$ can serve as the seed to generate the clock frequency by using, e.g., the direct digital synthesizer (DDS). The other is the transfer oscillator, which is, in principle, immune to the comb noise. Based on the electrical network, the optical frequency can be transferred into the microwave frequency with ultrahigh synchronicity. Figure 6 shows the optical setup on bench $1^{\prime }$ in SC1, in which the reference clock of ADCs is generated by the downconversion of the cavity-stabilized laser using the frequency comb. The other SCs hold the same configuration as this in SC1.

Here, we derive the TDI combinations with OFCs, which means that the clock noise is traceable to the laser frequency noise with a specific factor. As shown in Figure 3, there are six phase-locking schemes. Taking into account the practical operation, shorter link and more inner-spacecraft locking are preferable. Therefore, scheme C is recommended. Let us first consider scheme C. Please note that, in practice, considering the power consumption and the device size, it is recommended that one spacecraft takes one comb along. Consequently, assume that lasers $p_{1^{\prime }}$, $p_{2^{\prime }}$, and $p_{3}$ are used to generate the clocks in different SCs; $p_{1}$, $p_{2}$, and $p_{3^{\prime }}$ can be therefore expressed as

(15) $$\begin{array}{cc} q_{1}= & \Lambda_{1}p_{1^{\prime }}, \end{array}$$

(16) $$\begin{array}{cc} q_{2}= & \Lambda_{2}p_{2^{\prime }}, \end{array}$$

(17) $$\begin{array}{cc} q_{3^{\prime }}= & \Lambda_{3^{\prime }}p_{3}. \end{array}$$

where $\Lambda_{1},\Lambda_{2}$, and $\Lambda_{3^{\prime }}$ are the coefficients corresponding to the heterodyne frequencies. As shown in Figure 3 and Equation (6), three inner-spacecraft locking and two inter-spacecraft locking exist. In the inner-spacecraft phase-locking, we use the $z_{i}$ combinations as the error signal, which is

(18) $$\begin{array}{c} z_{1}=\frac{\tau_{1}-\tau_{1^{\prime }}}{2}=p_{1^{\prime }}-p_{1}-A_{1}p_{1^{\prime }}. \end{array}$$

where $A_{1}$ are the coefficients corresponding to the inner-spacecraft heterodyne frequencies. When the phase-locking loop is closed, $p_{1}$ can be therefore expressed as

(19) $$\begin{array}{c} p_{1}=p_{1^{\prime }}-A_{1}p_{1^{\prime }}. \end{array}$$

Similarly,

(20) $$\begin{array}{c} p_{2}=p_{2^{\prime }}-A_{2}p_{2^{\prime }}, \end{array}$$

(21) $$\begin{array}{c} p_{3^{\prime }}=p_{3}-A_{3^{\prime }}p_{3}. \end{array}$$

we use the beat note of the carrier as the error signal to feedback control the slave laser. For instance,

(22) $$\begin{array}{c} s_{2^{\prime }}^{c}=D_{3^{\prime }}p_{1}-p_{2^{\prime }}+\left(-D_{3^{\prime }}\Delta_{1}+\Delta_{2^{\prime }}\right)-B_{2^{\prime }}p_{2^{\prime }}+h_{2^{\prime }}+N_{2^{\prime }}^{opt}. \end{array}$$

where $B_{1}$ are the coefficients corresponding to the inter-spacecraft heterodyne frequencies. Then, with the closed locking loop, as in Equation (12), $p_{2^{\prime }}$ can be written as

(23) $$\begin{array}{c} p_{2^{\prime }}=\frac{1}{1+B_{2^{\prime }}}\left[D_{3^{\prime }}p_{1}+\left(-D_{3^{\prime }}\Delta_{1}+\Delta_{2^{\prime }}\right)+h_{2^{\prime }}+N_{2^{\prime }}^{opt}+N_{2^{\prime }}^{PLL}\right]. \end{array}$$

where the WLPL noise is involved.

Similarly, $p_{3}$ can be expressed as

(24) $$\begin{array}{c} p_{3}=\frac{1}{1+B_{3}}\left[D_{2}p_{1^{\prime }}+\left(-D_{2}\Delta_{1^{\prime }}+\Delta_{3}\right)+h_{3}+N_{3}^{opt}+N_{3}^{PLL}\right]. \end{array}$$

Based on Equations (19), (20), (22)–(24), we find that all the slave lasers can be traceable to the master laser $p_{1^{\prime }}$. We can obtain

(25) $$\begin{array}{cc} p_{1}= & p_{1^{\prime }}-A_{1}p_{1^{\prime }}, \end{array}$$

(26) $$\begin{array}{cc} p_{2^{\prime }}= & \frac{1}{1+B_{2^{\prime }}}\left[D_{3^{\prime }}\left(p_{1^{\prime }}-A_{1}p_{1^{\prime }}\right)+\left(-D_{3^{\prime }}\Delta_{1}+\Delta_{2^{\prime }}\right)+h_{2^{\prime }}+N_{2^{\prime }}^{opt}+N_{2^{\prime }}^{PLL}\right], \end{array}$$

(27) $$\begin{array}{cc} p_{2}= & \frac{1-A_{2}}{1+B_{2^{\prime }}}\left[D_{3^{\prime }}\left(p_{1^{\prime }}-A_{1}p_{1^{\prime }}\right)+\left(-D_{3^{\prime }}\Delta_{1}+\Delta_{2^{\prime }}\right)+h_{2^{\prime }}+N_{2^{\prime }}^{opt}+N_{2^{\prime }}^{PLL}\right], \end{array}$$

(28) $$\begin{array}{cc} p_{3^{\prime }}= & \frac{1-A_{3^{\prime }}}{1+B_{3}}\left[D_{2}p_{1^{\prime }}+\left(-D_{2}\Delta_{1^{\prime }}+\Delta_{3}\right)+h_{3}+N_{3}^{opt}+N_{3}^{PLL}\right], \end{array}$$

(29) $$\begin{array}{cc} p_{3}= & \frac{1}{1+B_{3}}\left[D_{2}p_{1^{\prime }}+\left(-D_{2}\Delta_{1^{\prime }}+\Delta_{3}\right)+h_{3}+N_{3}^{opt}+N_{3}^{PLL}\right]. \end{array}$$

We find that the laser frequency noise $p_{1^{\prime }}$ has been traveling along the arms. In addition, the spacecraft motion noise, the GW signal, the shot noise, and the WLPL noise are all transferred along the corresponding pathways as well. Here, we discuss the transfer features of these noises in detail. In the case of scheme C, the locking loop begins clockwise at $p_{3^{\prime }}$, and $p_{1^{\prime }}$ is the end point, anticlockwise at $p_{2}$, and $p_{1^{\prime }}$ is the end point. Therefore, we can directly analyze $p_{2}$ to clarify the noise transfer characteristics based on the polynomial coefficients in Equation (27). We find that the coefficients of $p_{1^{\prime }}$, and $\Delta_{1}$ are all $D_{3^{\prime }}$, and this means that the master laser frequency noise $p_{1^{\prime }}$ and the OB1 noise of $\Delta_{1}$ have been traveling along $L_{3^{\prime }}$. In addition, the OB $2^{\prime }$ noise is $\Delta_{2^{\prime }}$, the GW signal and the shot noise is $h_{2^{\prime }}$, and $N_{2^{\prime }}^{opt}$ is the local noise.From this simple example, it is clear that if the optical transponder scheme operates in the constellation, various noises, as well as the GW signals, will transfer along their own paths. Analogous with scheme C, the other schemes can be also analyzed based on the polynomial coefficients of the noises, which we do not discuss here due to the high similarity.

Next, the combined data streams, i.e., using scientific data streams, proof mass data streams, and reference data streams to eliminate spacecraft motion noise, can be expressed as

(30) $$\begin{array}{cc} \eta_{1^{\prime }}^{c}= & \left(D_{2^{\prime }2}\frac{1}{1+B_{3}}-1-B_{1^{\prime }}\right)p_{1^{\prime }}+\left(D_{2^{\prime }2}\frac{1}{1+B_{3}}+1\right)\delta_{1^{\prime }}-\left(D_{2^{\prime }}\frac{1}{1+B_{3}}+D_{2^{\prime }}\right)\delta_{3}\\ \; & +D_{2^{\prime }}\frac{1}{1+B_{3}}\left(h_{3}+N_{3}^{opt}\right)+h_{1^{\prime }}+N_{1^{\prime }}^{opt}+D_{2^{\prime }}\frac{1}{1+B_{3}}N_{3}^{PLL}, \end{array}$$

(31) $$\begin{array}{cc} \eta_{1}^{c}= & \left[D_{33^{\prime }}\frac{1}{1+B_{2^{\prime }}}\left(1-A_{1}\right)-\left(1-A_{1}\right)-B_{1}\right]p_{1^{\prime }}-\left(D_{33^{\prime }}\frac{1}{1+B_{2^{\prime }}}+1\right)\delta_{1}\\ \; & +\left(D_{3}\frac{1}{1+B_{2^{\prime }}}+D_{3}\right)\delta_{2^{\prime }}+D_{3}\frac{1}{1+B_{2^{\prime }}}\left(h_{2^{\prime }}+N_{2^{\prime }}^{opt}\right)+h_{1}+N_{1}^{opt}+D_{3}\frac{1}{1+B_{2^{\prime }}}N_{2^{\prime }}^{PLL}, \end{array}$$

(32) $$\begin{array}{cc} \eta_{2}^{c}= & \left[D_{12}\frac{1-A_{3^{\prime }}}{1+B_{3}}-\frac{1-A_{2}+B_{2}}{1+B_{2^{\prime }}}D_{3^{\prime }}\left(1-A_{1}\right)\right]p_{1^{\prime }}+D_{12}\frac{1-A_{3^{\prime }}}{1+B_{3}}\delta_{1^{\prime }}-D_{1}\frac{1-A_{3^{\prime }}}{1+B_{3}}\delta_{3}\\ \; & +\frac{1-A_{2}+B_{2}}{1+B_{2^{\prime }}}\left(D_{3^{\prime }}\delta_{1}-\delta_{2^{\prime }}\right)-\left(-D_{1}\delta_{3^{\prime }}+\delta_{2}\right)+D_{1}\frac{1-A_{3^{\prime }}}{1+B_{3}}\left(h_{3}+N_{3}^{opt}\right)\\ \; & -\frac{1-A_{2}+B_{2}}{1+B_{2^{\prime }}}\left(h_{2^{\prime }}+N_{2^{\prime }}^{opt}\right)+h_{2}+N_{2}^{opt}+D_{1}\frac{1-A_{3^{\prime }}}{1+B_{3}}N_{3}^{PLL}-\frac{1-A_{2}+B_{2}}{1+B_{2}}N_{2^{\prime }}^{PLL}, \end{array}$$

(33) $$\begin{array}{cc} \eta_{3^{\prime }}^{c}= & \left[D_{1^{\prime }3^{\prime }}\frac{1-A_{2}}{1+B_{2^{\prime }}}\left(1-A_{1}\right)-\frac{1-A_{3^{\prime }}+B_{3^{\prime }}}{1+B_{3}}D_{2}\right]p_{1^{\prime }}-D_{1^{\prime }3^{\prime }}\frac{1-A_{2}}{1+B_{2^{\prime }}}\delta_{1}\\ \; & +D_{1^{\prime }}\frac{1-A_{2}}{1+B_{2^{\prime }}}\delta_{2^{\prime }}+\frac{1-A_{3^{\prime }}+B_{3^{\prime }}}{1+B_{3}}\left(-D_{2}\delta_{1^{\prime }}+\delta_{3}\right)-\left(D_{1^{\prime }}\delta_{2}-\delta_{3^{\prime }}\right)\\ \; & +D_{1^{\prime }}\frac{1-A_{2}}{1+B_{2^{\prime }}}\left(h_{2^{\prime }}+N_{2^{\prime }}^{opt}\right)-\frac{1-A_{3^{\prime }}+B_{3^{\prime }}}{1+B_{3}}\left(h_{3}+N_{3}^{opt}\right)\\ \; & +h_{3^{\prime }}+N_{3^{\prime }}^{opt}+D_{1^{\prime }}\frac{1-A_{2}}{1+B_{2^{\prime }}}N_{2^{\prime }}^{PLL}-\frac{1-A_{3^{\prime }}+B_{3^{\prime }}}{1+B_{3}}N_{3}^{PLL}. \end{array}$$

We find that the WLPL noises still exist. As mentioned before, two additional data streams can be easily picked up in PLL, which are

(34) $$\begin{array}{cc} s_{2^{\prime }}^{er}= & {-N}_{2^{\prime }}^{PLL}\left(t\right) \end{array}$$

(35) $$\begin{array}{cc} s_{3}^{er}= & {-N}_{3}^{PLL}\left(t\right) \end{array}$$

The WLPL noises in Equations (30)–(33) are exactly the delayed version of the two data streams. Consequently, the WLPL noises can be efficiently removed by the combinations

(36) $$\begin{array}{cc} \eta_{1^{\prime }}^{PLL}= & \;\eta_{1^{\prime }}^{c}+D_{2^{\prime }}\frac{1}{1+B_{3}}s_{3}^{er}\\ = & \left(D_{2^{\prime }2}\frac{1}{1+B_{3}}-1-B_{1^{\prime }}\right)p_{1^{\prime }}+\left(D_{2^{\prime }2}\frac{1}{1+B_{3}}+1\right)\delta_{1^{\prime }}\\ \; & -\left(D_{2^{\prime }}\frac{1}{1+B_{3}}+D_{2^{\prime }}\right)\delta_{3}+D_{2^{\prime }}\frac{1}{1+B_{3}}\left(h_{3}+N_{3}^{c}\right)+h_{1^{\prime }}+N_{1^{\prime }}^{opt}, \end{array}$$

(37) $$\begin{array}{cc} \eta_{1}^{PLL}= & \;\eta_{1}^{c}+D_{3}\frac{1}{1+B_{2^{\prime }}}s_{2^{\prime }}^{er}\\ = & \;\left[D_{33^{\prime }}\frac{1}{1+B_{2^{\prime }}}\left(1-A_{1}\right)-\left(1-A_{1}\right)-B_{1}\right]p_{1^{\prime }}-\left(D_{33^{\prime }}\frac{1}{1+B_{2^{\prime }}}+1\right)\delta_{1}\\ \; & +\left(D_{3}\frac{1}{1+B_{2^{\prime }}}+D_{3}\right)\delta_{2^{\prime }}+D_{3}\frac{1}{1+B_{2^{\prime }}}\left(h_{2^{\prime }}+N_{2^{\prime }}^{opt}\right)+h_{1}+N_{1}^{opt}, \end{array}$$

(38) $$\begin{array}{cc} \eta_{2}^{PLL}= & \;\eta_{2}^{c}+D_{1}\frac{1-A_{3^{\prime }}}{1+B_{3}}s_{3}^{er}-\frac{1-A_{2}+B_{2}}{1+B_{2}}s_{2^{\prime }}^{er}\\ = & \;\left[D_{12}\frac{1-A_{3^{\prime }}}{1+B_{3}}-\frac{1-A_{2}+B_{2}}{1+B_{2^{\prime }}}D_{3^{\prime }}\left(1-A_{1}\right)\right]p_{1^{\prime }}+D_{12}\frac{1-A_{3^{\prime }}}{1+B_{3}}\delta_{1^{\prime }}-D_{1}\frac{1-A_{3^{\prime }}}{1+B_{3}}\delta_{3}\\ \; & +\frac{1-A_{2}+B_{2}}{1+B_{2^{\prime }}}\left(D_{3^{\prime }}\delta_{1}-\delta_{2^{\prime }}\right)-\left(-D_{1}\delta_{3^{\prime }}+\delta_{2}\right)+D_{1}\frac{1-A_{3^{\prime }}}{1+B_{3}}\left(h_{3}+N_{3}^{opt}\right)\\ \; & -\frac{1-A_{2}+B_{2}}{1+B_{2^{\prime }}}\left(h_{2^{\prime }}+N_{2^{\prime }}^{opt}\right)+h_{2}+N_{2}^{opt}, \end{array}$$

(39) $$\begin{array}{cc} \eta_{3^{\prime }}^{PLL}= & \;\eta_{3^{\prime }}^{c}+D_{1^{\prime }}\frac{1-A_{2}}{1+B_{2^{\prime }}}s_{2^{\prime }}^{er}-\frac{1-A_{3^{\prime }}+B_{3^{\prime }}}{1+B_{3}}s_{3}^{er}\\ = & \left[D_{1^{\prime }3^{\prime }}\frac{1-A_{2}}{1+B_{2^{\prime }}}\left(1-A_{1}\right)-\frac{1-A_{3^{\prime }}+B_{3^{\prime }}}{1+B_{3}}D_{2}\right]p_{1^{\prime }}\\ \; & -D_{1^{\prime }3^{\prime }}\frac{1-A_{2}}{1+B_{2^{\prime }}}\delta_{1}+D_{1^{\prime }}\frac{1-A_{2}}{1+B_{2^{\prime }}}\delta_{2^{\prime }}+\frac{1-A_{3^{\prime }}+B_{3^{\prime }}}{1+B_{3}}\left(-D_{2}\delta_{1^{\prime }}+\delta_{3}\right)\\ \; & -\left(D_{1^{\prime }}\delta_{2}-\delta_{3^{\prime }}\right)+D_{1^{\prime }}\frac{1-A_{2}}{1+B_{2^{\prime }}}\left(h_{2^{\prime }}+N_{2^{\prime }}^{opt}\right)-\frac{1-A_{3^{\prime }}+B_{3^{\prime }}}{1+B_{3}}\left(h_{3}+N_{3}^{opt}\right)+h_{3^{\prime }}+N_{3^{\prime }}^{opt}. \end{array}$$

From Equations (36)–(39), we find that $\eta_{i}^{PLL}$ and $\eta_{i^{\prime }}^{PLL}$ only contain the laser frequency noise $p_{1^{\prime }}$, the proof mass noise, the GW signals, and the shot noise. We can easily obtain a series of TDI combinations, and the combination $H_{1}$ using $\eta_{1}^{PLL}$ and $\eta_{1^{\prime }}^{PLL}$ is

(40) $$\begin{array}{cc} H_{1}^{FC}= & \left(D_{33^{\prime }}\frac{1-A_{1}}{1+B_{2^{\prime }}}-\left(1-A_{1}\right)-B_{1}\right)\eta_{1^{\prime }}^{PLL}\\ \; & -\left(D_{2^{\prime }2}\frac{1}{1+B_{3}}-1-B_{1^{\prime }}\right)\eta_{1}^{PLL}. \end{array}$$

If we consider that the delay operators are commuting, the laser frequency noise can be canceled out based on Equation (40). We find that by using the frequency comb, the laser frequency noise, WLPL noise, and the clock noise can be reduced by one step of TDI simultaneously.

Please note that we can actually derive six kinds of TDI combinations based on Equations (36)–(39), which are the combinations of $H_{1}$ (using $\eta_{1^{\prime }}^{PLL}$ and $\eta_{1}^{PLL}$), $H_{2}$ (using $\eta_{1^{\prime }}^{PLL}$ and $\eta_{2}^{PLL}$), $H_{3}$ (using $\eta_{1^{\prime }}^{PLL}$ and $\eta_{3^{\prime }}^{PLL}$), $H_{4}$ (using $\eta_{1}^{PLL}$ and $\eta_{2}^{PLL}$), $H_{5}$ (using $\eta_{1}^{PLL}$ and $\eta_{3^{\prime }}^{PLL}$), and $H_{6}$ (using $\eta_{2}^{PLL}$ and $\eta_{3^{\prime }}^{PLL}$), respectively. In this subsection, we analyze the $H_{1}$ combination in detail, and do not give the description of the other combinations with consideration of the resemblance and the article length.

## 5. Time-Domain Simulation

**Figure 7:**
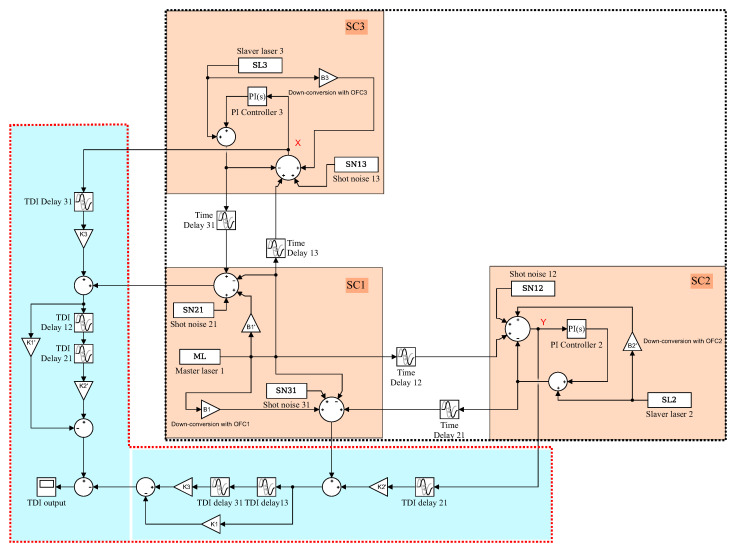
The block diagram of the time-domainsimulation in Simulink. SC: spacecraft.

**Figure 8:**
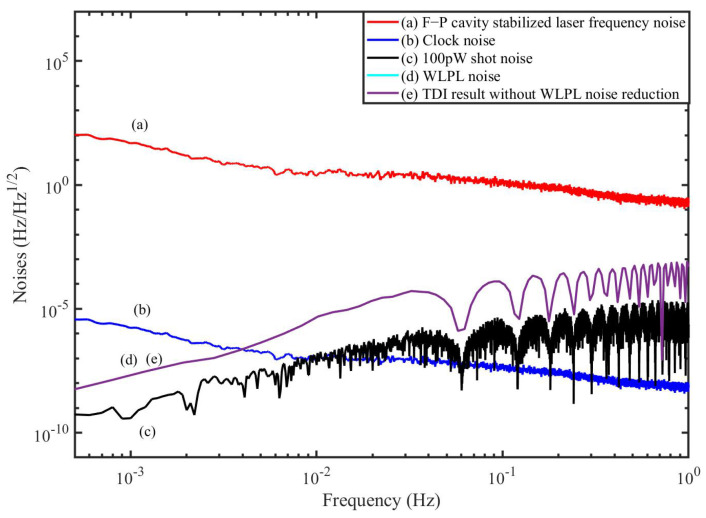
TDI results of the simulation without WLPL noise reduction.

In this section, we perform the time-domain simulation to examine the performance of the TDI combination. The block diagram is depicted in Figure 7, which is actually composed of two parts. One part (in the black dashed box) is the inter-spacecraft optical interferometer, and the other (in the red dashed box) is the module of the TDI process. In the optical interferometer, the delays between the spacecraft are 8.3 s and 8.4 s, respectively. The WLPL is based on the PI controller, and the optical power of the weak light is 100 pW. We measure the noises of the FP-stabilized laser (homemade with ULE cavity, 1064 nm [31]) and WLPL noise in our lab, as shown in Figure 8, and inject the noises into the simulation. The clock noises are generated by the downconversion of the laser frequency noises with the factor of about 10 MHz/282 THz, while the repetition frequency of the frequency comb is about 250 MHz. The shot noise is calculated by ${\left(\frac{\hbar c}{2\pi }\frac{1}{\lambda P_{d}}\right)}^{1/2}f$, where *f* is the Fourier frequency, λ is the laser wavelength, and $P_{d}$ is the optical power of the weak light. As mentioned before, the WLPL noise is the slight difference between the frequencies of the master and slave lasers, and can be extracted via the error signals in the phase-locking loop. In the simulation, the WLPL noise can be directly picked up at points X and Y.

**Figure 9:**
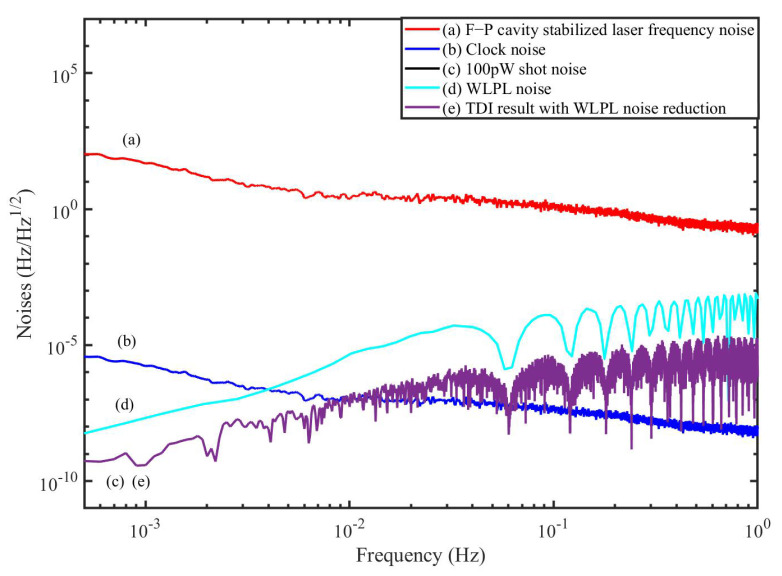
TDI results of the simulation with WLPL noise reduction.

Figure 8 shows the simulation results without the reduction of the WLPL noise. We find that the laser frequency noise can be reduced, but is not able to reach the level of the shot noise. This is because the residual WLPL noise exists in the system. In contrast, if we consider the WLPL noise and use the combinations in Equation (40), the results are shown in Figure 9. We find that the results after TDI can be improved to the limit of the shot noise. Our results show that the WLPL noise can be reduced by the technique of TDI. Note that the WLPL noise and the shot noise in Figure 5 are different from those in Figure 8 and Figure 9. This is because in TDI post-processing, all the data are multiplied by a transfer function due to the time delay [32], which is $1-e^{-s\tau }$, where *s* is complex frequency (or Laplace variable), and τ is the delay time. We can find that this transfer function has multiple zeros at Fourier frequency of f=n/τ, and *n* is an integer.

## 6. Discussion

In the discussion, we only analyze one kind of TDI combination in scheme C in detail. A wealth of other combinations in other locking schemes are not described due to the high resemblance. On the other hand, if we consider the real situations, schemes A, C, and E are recommended because only two inter-spacecraft locking loops are involved. For schemes B, D, and F, there are three weak-light locking loops. Additionally, shorter locking link is more reliable in practice. Therefore, scheme C is more preferable.

## 7. Conclusions

In conclusion, we derive the phase-locking TDI combinations with OFCs with consideration of the reduction of the WLPL noise. We show that the unique combination of phase-locking and OFCs can greatly simplify the TDI combinations. With the help of OFCs, an ultrastable oscillator based on the optic-to-microwave link can be generated, which can serve as the time base in the spacecraft. In this case, the performance of the ultrastable oscillator is determined by the cavity-stabilized laser. It is necessary to point out that the noise of the clock generated with the OFC can be less than $10^{-6}\;\mathrm{Hz}/{\mathrm{Hz}}^{1/2}$ in the science band if the laser frequency noise can be better than $10\;\mathrm{Hz}/{\mathrm{Hz}}^{1/2}$. This level of performance is not easy to reach for the crystal oscillators. Because the clock noise has been synchronized to the laser frequency noise, one single step of TDI can realize the simultaneous reduction of laser frequency noise and clock noise. In actuality, the whole constellation will share not only one common laser, but also one common clock, giving rise to a significant simplification in the post-processing of TDI measurements. Limited by the performance of the weak-light phase-locking loop, the WLPL noise above the noise floor would be involved, and cannot be neglected. We demonstrate that the WLPL noise can be well reduced by using the error signals in the TDI process. Consequently, the requirement of the WLPL would be relaxed in future. Many noises, such as the spacecraft motion noise, the shot noise, and WLPL noise, will travel along the long arms following the specific paths. The transfer features were analyzed and various TDI combinations can be obtained. Finally, the time-domain simulation was carried out to examine the performance of the TDI combination by using the measurement results in our lab. The results show that the laser frequency noise and the clock noise can be well suppressed simultaneously using the TDI combination with frequency comb. The WLPL noise can be also reduced with the error signals involved in the TDI combination. To date, the reliable operation of frequency combs has been reported [33], showing that laser frequency combs can serve in future space missions. Nevertheless, the device size, weight, and power consumption of the combs should be carefully considered due to the limited resources in the spacecraft. In the future space-borne GW detectors, we suggest that the microcombs would be the qualified candidates because of their compact device footprint and low power consumption. Our work focuses on the phase-locking characteristics with OFCs, and could offer a valuable proposal for future phase-locking time-delay interferometry.

## Data Availability

The data can be obtained upon reasonable request to the corresponding author.
